# The Dynamics of Metabolic Characterization in iPSC-Derived Kidney Organoid Differentiation *via* a Comparative Omics Approach

**DOI:** 10.3389/fgene.2021.632810

**Published:** 2021-02-10

**Authors:** Qizheng Wang, Yucui Xiong, Sheng Zhang, Yufei Sui, Cunlai Yu, Peng Liu, Heying Li, Wenjing Guo, Yubo Gao, Aneta Przepiorski, Alan J. Davidson, Meijin Guo, Xiao Zhang

**Affiliations:** ^1^State Key Laboratory of Bioreactor Engineering, East China University of Science and Technology, Shanghai, China; ^2^CAS Key Laboratory of Regenerative Biology, Joint School of Life Sciences, Guangzhou Institutes of Biomedicine and Health, Chinese Academy of Sciences, Guangzhou Medical University, Guangzhou, China; ^3^Bioland Laboratory (Guangzhou Regenerative Medicine and Health Guangdong Laboratory), Guangzhou, China; ^4^Department of Urology, Nanfang Hospital, Southern Medical University, Guangzhou, China; ^5^Department of Molecular Medicine and Pathology, University of Auckland, Auckland, New Zealand

**Keywords:** induced pluripotent stem cells, kidney organoids, transcriptomics, metabolomics, serine metabolism

## Abstract

The use of differentiating human induced pluripotent stem cells (hiPSCs) in mini-tissue organoids provides an invaluable resource for regenerative medicine applications, particularly in the field of disease modeling. However, most studies using a kidney organoid model, focused solely on the transcriptomics and did not explore mechanisms of regulating kidney organoids related to metabolic effects and maturational phenotype. Here, we applied metabolomics coupled with transcriptomics to investigate the metabolic dynamics and function during kidney organoid differentiation. Not only did we validate the dominant metabolic alteration from glycolysis to oxidative phosphorylation in the iPSC differentiation process but we also showed that glycine, serine, and threonine metabolism had a regulatory role during kidney organoid formation and lineage maturation. Notably, serine had a role in regulating S-adenosylmethionine (SAM) to facilitate kidney organoid formation by altering DNA methylation. Our data revealed that analysis of metabolic characterization broadens our ability to understand phenotype regulation. The utilization of this comparative omics approach, in studying kidney organoid formation, can aid in deciphering unique knowledge about the biological and physiological processes involved in organoid-based disease modeling or drug screening.

## Introduction

Personalized induced pluripotent stem cells (iPSCs) is an emerging technology that increases the number of options available for regenerative medicine applications (Robinton and Daley, 2012; Cossu et al., 2018). A major advance in recent years has been the use of stem cell-derived organoids as *in vitro* models to study development and disease (Lancaster and Knoblich, 2014). The ability of iPSCs to differentiate into various cell types has been utilized recently to generate organoids (Wiegand and Banerjee, 2019). Organoid culture has been established for various organs and tissues, including the intestine, heart, liver, brain, and kidney (Kretzschmar and Clevers, 2016).

Unlike zebrafish, which have a single unit of the nephron that can be repaired or regenerated to a certain extent (Diep et al., 2011; Kroeger and Wingert, 2014), the human kidney contains about 1–1.5 million nephrons serving as the functional units within each kidney. Hence, generating complex kidney tissues, such as kidney organoids, in a large quantity from personalized human induced pluripotent stem cells (hiPSCs) have broadened our ability to study human kidney development, disease, and even perform tissue engineering (Xia et al., 2013; Taguchi et al., 2014; Takasato et al., 2014, 2015; Freedman et al., 2015; Morizane et al., 2015; Taguchi and Nishinakamura, 2017; Przepiorski et al., 2018; Low et al., 2019). These methods have established a concrete foundation for generating kidney organoids through the modulation of wingless-related integration site (WNT), fibroblast growth factor (FGF), and transforming growth factor β (TGF-β) signaling. Although the mechanisms involved in the different protocols have been well-studied, iPSCs derived from different donors perform differently between similar protocols. Additionally, batch variation was not accounted for, and during the culture process, quality feedback from the derived kidney organoids was not performed adequately.

Emerging studies of stem cell metabolism have demonstrated that cellular metabolic pathways influence the proliferation, reprogramming, or differentiation process (Zhang et al., 2012, 2018; Xu et al., 2013; Ryall et al., 2015). Monitoring the metabolic profile is an effective way to describe the iPSC status because there are dynamic changes in mitochondrial morphology and metabolic shifts under different culture conditions (Shyh-Chang et al., 2013; Son et al., 2013; Wanet et al., 2015). Moreover, iPSC differentiation to other cell types is generally accompanied by cellular metabolism alterations. For example, a high glycolytic level stimulates embryonic stem cell (ESC) proliferation (Kim et al., 2006), whereas differentiating ESCs switch to oxidative phosphorylation (OXPHOS) (Folmes et al., 2012). The metabolic switch from OXPHOS to glycolysis occurs during the reprogramming process and cooperates with the epithelial-mesenchymal transition to regulate pluripotency (Sun et al., 2020a). In contrast, the bioenergetic profile changes from glycolysis to OXPHOS during cardiomyogenic differentiation (Lopaschuk and Jaswal, 2010), neuronal differentiation (Zheng et al., 2016), and induction of hepatocyte maturation (Hopkinson et al., 2017). Consistently, during the maturation process, nephron progenitor cells (NPCs) have significantly higher glycolysis compared with matured NPCs, and the inhibition of glycolysis promotes nephrogenesis in embryonic kidneys (Liu et al., 2017).

Therefore, we hypothesized that characterizing the metabolic profile of the kidney organoids derived from iPSCs might facilitate the discovery of the essential metabolic element. Metabolic profiling can be a more direct method to monitor the quality of organoid differentiation and maturation, and it can also describe the dynamic changes in cellular status during drug screening. Here, in this study, we used comparison studies between transcriptomics and untargeted metabolomics. The identification and correlation of time-dependent metabolic changes in iPSC-derived kidney organoids and the metabolic regulatory mechanism during differentiation were established. Furthermore, we identified that glycine, serine, and threonine metabolism might play an essential role during kidney organoid differentiation. Finally, we verified that during the kidney organoid differentiation, observation of DNA methylation enhancement was mediated through the regulation of serine metabolism-related bioactive metabolite (SAM).

## Results

### Generation of Human iPSC-Derived Kidney Organoids

**FIGURE 1:**
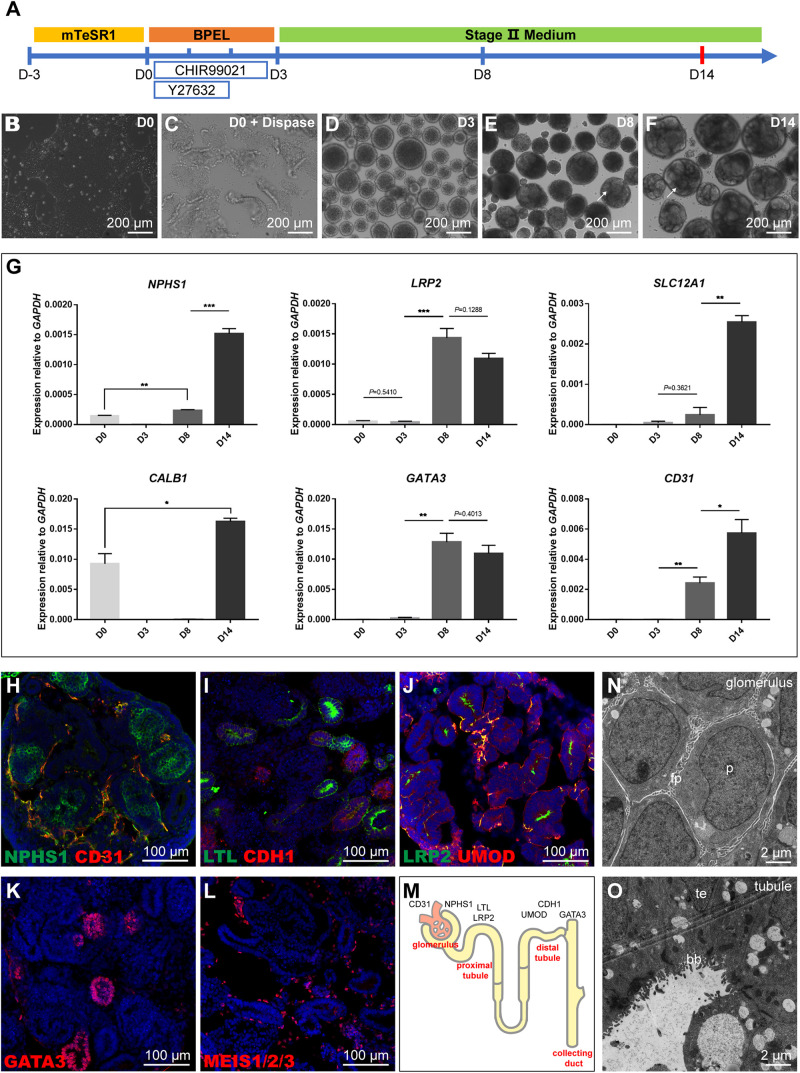
Schematic representation and characterization of the kidney organoid differentiation process. **(A)** Roadmap of kidney organoids derived from hiPSCs. **(B,C)** Starting iPSC colonies and iPSC colonies digested by dispase. **(D)** Embryoid bodies. **(E,F)** The phenotype of kidney organoids at day 8 (D8) and day 14 (D14); formation of tubules is visible on the surface (arrows). **(G)** qRT-PCR analysis for selected markers during the differentiation at day 0, 3, 8, and 14. Data are presented as mean ± SEM from biological triplicates. **P < 0.01, ***P < 0.001. **(H–L)** Immunostaining of frozen sections of day 14 organoids showing NPHS1+ podocytes and CD31+ endothelial cells, LTL+ proximal tubules, and CDH1+ distal tubules, LRP2+ proximal tubules, and UMOD+ thick ascending limb segments, GATA3+ collecting duct structures, and MEIS1/2/3+ interstitial cells. Nuclear counterstain: DAPI. **(M)** Schematic representation of kidney organoids (day 14). **(N,O)** Transmission electron micrograph (TEM) images of day 14 organoid sections: podocytes (p, podocytes; fp, primitive foot processes); and tubular (te, tubular epithelium; bb, brush border).

To obtain kidney organoids in a scalable manner, we modified a protocol from Przepiorski et al. (2018). The detailed procedure was explained in the methods, and the three stepwise differentiation procedure was illustrated (Figure 1A). Briefly, the assay’s initial setup involved growing hiPSC colonies in a monolayer to about 80% confluence. On day 0 (D0), the colonies were detached with dispase, then scraped and transferred into a 6-well ultra-low attachment plate for suspension culture to form aggregated embryoid bodies (EBs) (Figures 1B,C). Next the formed EBs were cultured in “Stage II” medium from day 3 (D3) until tubule formation which was visible under bright-field microscopy after day 8 (D8), and tubule structuralized organoids at around day 14 (D14) (Figures 1D–F). Subsequently, the quantification of gene expression for lineage markers such as NPHS1 (podocytes), LRP2 (proximal tubule), SLC12A1 (thick ascending limb), CALB1 (collecting duct), GATA3 (collecting duct), and CD31 (endothelial cells) was carried out at different time points using quantitative RT-PCR (qRT-PCR) (Figure 1G). The quantification showed that all these lineage or cell type-specific markers were upregulated from D0 to D14, which recapitulated the developmental program of nephrogenesis. Tubule formation might take a different lineage approach depending on differentiation condition, hence, apart from GATA3 (collecting duct) and LRP2 (proximal tubule) which had biphasic responses, all other markers had continual increases during the assays with the highest expression levels detected at D14.

Furthermore, immunostaining analysis of kidney organoids (D14) with a range of physiology-related markers also confirmed the results observed during nephron differentiation. The identification of NPHS1+ podocytes and CD31+ endothelial cells showed that kidney organoids contained glomerular structures and were surrounded by immature vascular cells (Figure 1H). Proximal segments that stained for Lotus tetragonolobus lectin (LTL) and distal portions labeled with cadherin-1 (CDH1) demonstrated the formation of the proximal and distal tubules (Figure 1I). The UMOD+ thick ascending limb segments were identified with a short segment existed between LRP2+ proximal tubules, which could be a part of the loop of Henle (Figure 1J); GATA3 had positive staining for collecting duct structures (Figure 1K); MEIS1/2/3+ interstitial cells were also identified that were scattered throughout the tissue (Figure 1L). These data collectively displayed that organoids (D14) consist of podocytes in the glomerular structures, proximal tubular, distal tubular, collecting duct structures, and interstitial cells (Figure 1M).

The maturation status was further determined by examining the glomerular and tubular structures using transmission electron micrograph (TEM) analysis. TEM results demonstrated characteristic structures, such as podocytes with primitive foot processes in the glomerulus (Figure 1N) and tubular structures with multi-layered epithelial structures and brush-borders in organoids at D14 (Figure 1O). Together, these results confirmed that we established a differentiated kidney organoid culture system consistent with the maturation levels published in the literature.

### Transcriptional Profiling Reveals the Metabolic Alteration That Occurs During Kidney Organoid Differentiation

**FIGURE 2:**
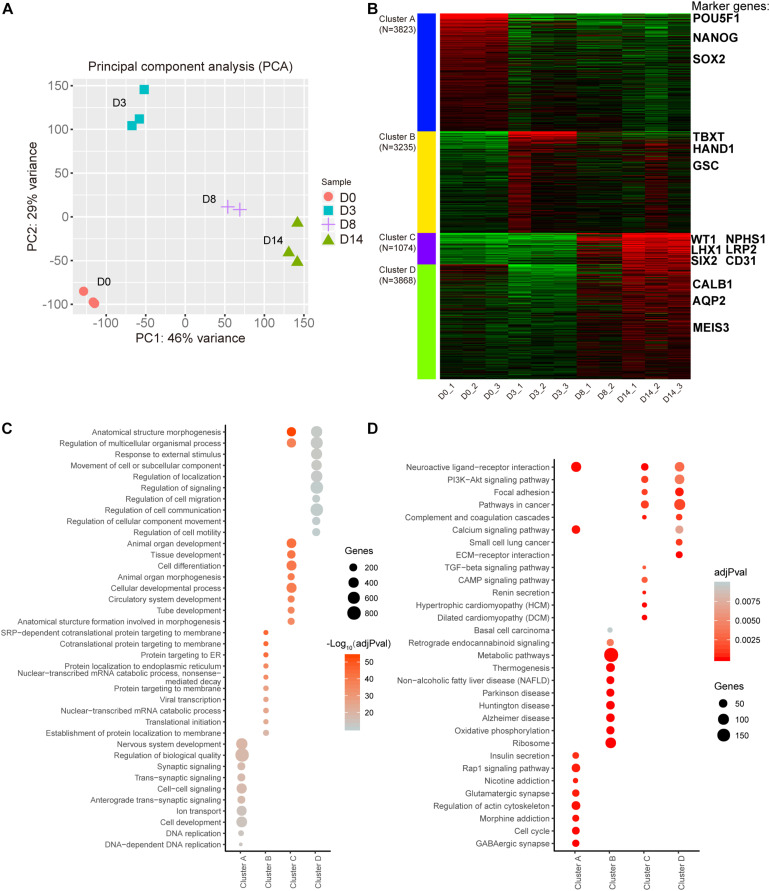
Transcription profiling of the kidney organoid differentiation process. **(A)** Scores plot for transcriptome samples in the PCA analysis. **(B)** k-Means clustering of expressed genes during different differentiation phases (the top 12,000 variable genes were used). Known markers of kidney development are highlighted. **(C)** Top 10 overrepresented GO terms in each cluster with functions in biological processes. **(D)** Top 10 enriched pathways in each cluster based on KEGG pathway enrichment analysis. Pathways with adjusted *P*-value (adjPval) below 0.01 (2C and 2D).

To understand the gene expression dynamics during the different stages of organoid differentiation, RNA-seq analysis of organoids was conducted at different time-lapse sampling points. A total of 11 samples from four different time points were sequenced for RNA expression (D8 group had only *n* = 2 biological repeats because of sequence library construction, the other groups had *n* = 3 repeats). According to Principal Component Analysis (PCA) based on counts per million (CPM) values of genes, replicate samples from similar time points were clustered closely (Figure 2A). These data illustrated that the correlation coefficients of samples were achieved (Supplementary Figure 1).

Subsequently, a k-Means clustering analysis was carried out, which divided the top 12,000 of the most variable genes ranked by the standard deviation into four clusters. Interestingly, each cluster had specific marker genes referring to different stages of nephrogenesis. In detail, POU5F1, NANOG, and SOX2 served as pluripotency markers in cluster A and were highly expressed in D0 organoids as expected. Simultaneously, mesoderm markers TBXT, HAND1, and GSC in cluster B were enriched in D3 organoids. Additionally, cluster C had both intermediate mesoderm markers (WT1, LHX1, and SIX2) and mature markers (NPHS1, LRP2, and CD31) enriched in D8 to D14 organoids, while cluster D had additional mature markers (CALB1, AQP2, and MEIS3) enriched in D14 organoids (Figure 2B and Supplementary Figure 2). These findings indicated that kidney organoids progressed through the mathematically-grouped clusters. As indicated, this grouping matched up with the development-related stages, which indicated maturation of renal cell types and correlated with the varied sampling times.

Furthermore, Gene Ontology (GO) biological progress annotation was exploited for each cluster gene (Figure 2B), which demonstrated that each cluster gene had a functional role in different biological processes. In particular, genes within cluster A were enriched for DNA replication and cell development progress. In contrast, genes within cluster B were significantly enriched in protein translation-related progression, while cluster C genes were related to the processes involved in differentiation, such as tissue development, tube development, and animal organ morphogenesis (Figure 2C).

Additionally, we performed Kyoto Encyclopedia of Genes and Genomes (KEGG) enrichment analysis for each cluster gene (Figure 2B) and found that 190 genes in cluster B were strikingly enriched in the metabolic pathway (Figure 2D). These data implied that metabolic gene expression dynamics appeared to have distinct profile stages during the differentiation of kidney organoids, which was consistent with our hypothesis that there might be a correlation between metabolic changes and progression of differentiation.

### Classification of Metabolic Genes and Changes in Metabolite Dynamics

**FIGURE 3:**
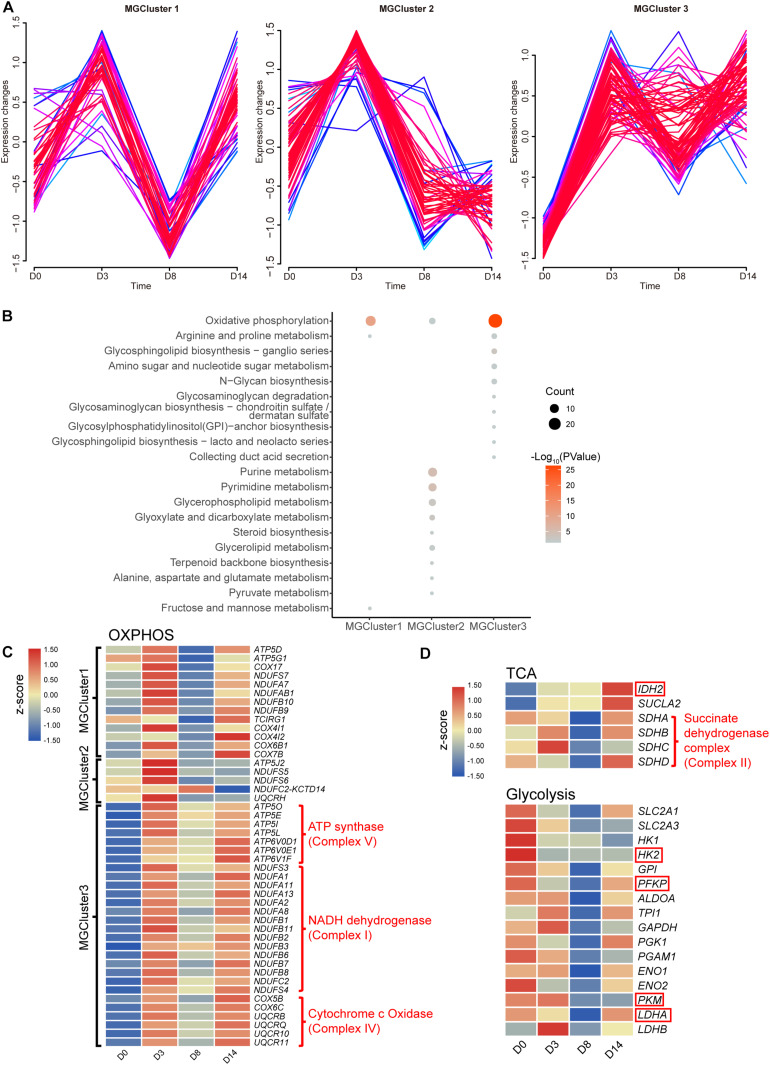
Dynamic changes in metabolite-related genes. **(A)** Time-course expression profiles of 190 genes enriched in the KEGG term metabolic pathways. **(B)** KEGG functional analysis of three overrepresented clusters. Pathways with a *P*-value below 0.05 are shown. **(C)** Heatmap of relative gene expression related to OXPHOS from three overrepresented clusters. **(D)** Heatmap of relative gene expression in the TCA cycle related to OXPHOS and in the glycolysis pathway. Genes highlighted in the red frame encoding the rate-limiting enzymes. Heatmap shows averaged values from biological replicates.

As reported in the literature, cellular metabolism can play crucial roles in maintaining pluripotency, differentiation, and reprogramming of iPSCs (Zhang et al., 2018). A metabolic switch from the glycolysis to OXPHOS state was described consistently in cells that differentiated from pluripotent states initially. Hence, we further investigated the perturbation of the intracellular metabolism stimulated by kidney organoid differentiation. The expression pattern of 190 genes enriched in the metabolic pathway from cluster B (Figure 2D) was divided into three clusters (MGCluster, R Mfuzz package), which included 46, 59, or 85 upregulated genes from D0 to D3, respectively, corresponding to three diverse expression patterns (Figure 3A). MGCluster 1 contained downregulated genes at D8 but upregulated at D14, and genes in MGCluster 2 declined continuously from D3 to D14, while the expression of genes overrepresented in MGCluster 3 remained at a relatively high level from D3 to D14.

Subsequently, we performed KEGG pathway enrichment analysis to characterize the function of these three MGClusters (Figure 3B). Consistently, we found that every MGCluster was significantly enriched in OXPHOS, indicating the dynamic transcriptional response of OXPHOS-related genes involved in kidney organoid differentiation, which were associated with the accelerated generation of intracellular energy. This finding confirmed the relative expression level of genes enriched in OXPHOS during the progression of kidney organoid differentiation (Figure 3C). MGCluster 3 contained the major complexes that encoded genes in the OXPHOS. Incorporating genes were upregulated consistently during differentiation and this was the dominant dynamic pattern among the three clusters.

We paid particular attention to the expression levels of *SDH*, *IDH2* encoding isocitrate dehydrogenase (which is regarded as a rate-limiting enzyme in the tricarboxylic acid (TCA) cycle), and *SUCLA2* encoding succinyl-CoA synthetase beta subunit, because of their essential role in the TCA cycle or OXPHOS. The gene expression level was verified using qRT-PCR *via* the selected marker *IDH2* (Supplementary Figure 3). The verified RNA-seq data were plotted in the heatmap and this demonstrated that the transcriptional level of *SUCLA2* and *IDH2* consistently improved. This improvement possibly benefited from the provision of more reducing equivalents and an energy substrate for OXPHOS by elevating the carbon flux through the TCA cycle (Figure 3D). Moreover, the altered expression level of *SDH* could provide alternative evidence for the activation of OXPHOS during the later phases of differentiation.

To systematically characterize and evaluate the metabolic changes during transcriptional regulation, we simultaneously performed untargeted metabolomic analysis to identify key metabolic profiles. PCA analysis demonstrated that samples from all four-time points were separated into four distinct groups, as shown in the scores plot (Supplementary Figure 4A), suggesting a recognizable alteration in metabolism during the different stages of differentiation. The heatmap of 96 annotated metabolites among different samples is shown in Supplementary Figure 4B. The change of pool sizes of these metabolites could also be divided into three clusters (Supplementary Figure 4C), and the result of KEGG functional analysis was shown (Supplementary Figure 4D).

Accordingly, several metabolic pathways held a consistent alteration pattern on both a transcriptional and metabolic level, these include purine, pyrimidine, alanine, aspartate, and glutamate metabolism (Supplementary Figure 5), which might associate with a high requirement of nucleotides in the early stage of differentiation (from D0 to D3). Collectively, to a certain extent, these data demonstrated that alterations in metabolism measured directly in this study were consistent with the transcriptional levels of enriched genes, which served as the cross-references to verify these findings.

### Integrative Transcriptomic and Metabolomic Analysis Reveals the Consistency of Glycolysis and TCA Cycle Pathway Changes During Kidney Organoid Differentiation

**FIGURE 4:**
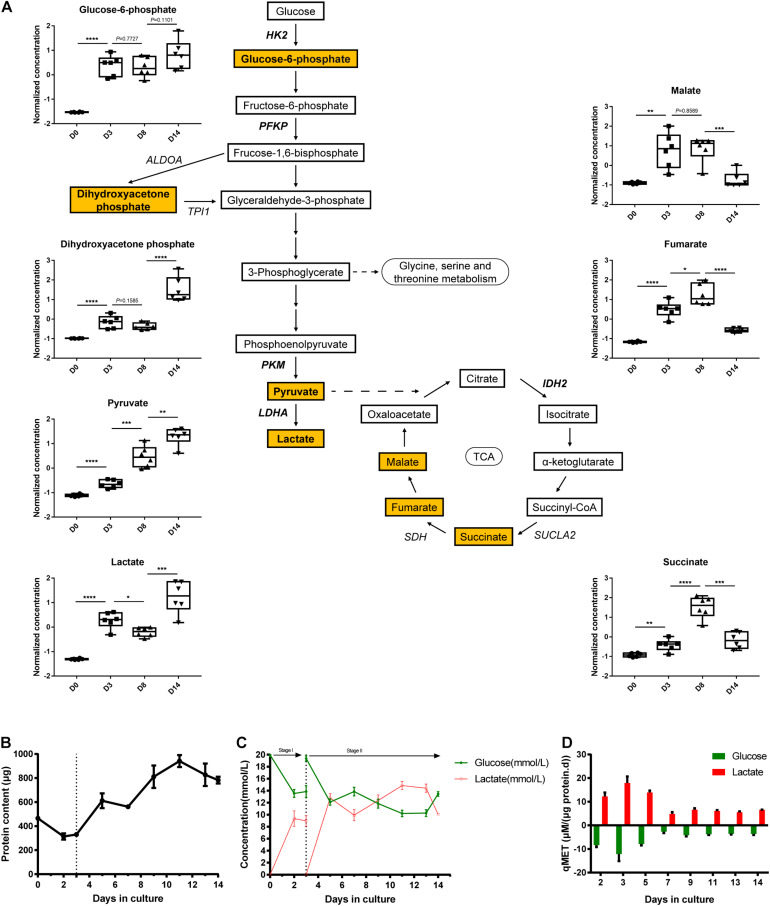
Transcriptional and metabolic changes in glycolysis and TCA cycle. **(A)** Schematic of the glycolysis pathway and TCA cycle, the normalized and auto-scaled concentration of quantified metabolites are highlighted. **(B)** The total protein content of cells during the cultivation. **(C)** Concentration of extracellular residual glucose (green line) and lactate (red line) during differentiation. **(D)** Specific consumption rates of glucose (green column) and specific generation rate of lactate (red column) at different time points. The normalization of concentration of metabolites is described in methods. Data are presented as mean ± SEM from at least three biological replicates. *P < 0.05, **P < 0.01, ***P < 0.001, and ****P < 0.0001.

To determine glycolysis and TCA cycle metabolic profiles, we co-analyzed the transcriptomics data combined with the metabolomics data. We measured several glycolytic and TCA metabolites using liquid chromatography-mass spectrometry (LC-MS), including glucose-6-phosphate, dihydroxyacetone phosphate, pyruvate, lactate, malate, fumarate, and succinate (Figure 4A). As shown in Figure 3D, the expression of the majority of glycolytic genes decreased from D0 to D14, which could be related to the inhibited rate-limiting enzymes in glycolysis, such as *HK2* and *PKM*. The finding showed that the four measured glycolytic metabolites (glucose-6-phosphate, dihydroxyacetone phosphate, pyruvate, and lactate) detected in pathway activity were determined in a consumption-related manner. These measured glycolytic metabolites illustrated the moderately low levels in D0, which were correlated with metabolite consumption, which then increased during the late phase of organoid differentiation.

On the contrary, the expression patterns of genes elucidated the potentiation of TCA cycle activation from D0 to D14 (Figure 3D). Consistently, the abundance of several TCA cycle intermediates (malate, fumarate, and succinate as detected) was elevated from D0 and peaked at D8. This finding implied the redistribution of metabolic flux from the glycolysis pathway toward the TCA cycle during the differentiation process. Despite the continuous increase in the expression level of these three metabolites, the pool sizes of the three TCA cycle intermediates (listed above) declined at D14 strikingly. This observation can be explained as the enhancement of turnover during TCA cycle activation.

As a comparison study, we performed direct biochemical measurement of the consumption rate of glucose and the accumulation rate of lactate in the culture medium throughout the 14-day differentiation process. Moreover, cell proliferation in the early stage of kidney organoid differentiation was observed (data not shown), which was in agreement with the raised total protein content for quantifying the biomass (Figure 4B). Further, the specific carbon uptake rate and lactate generation rate both remarkably declined during the differentiation, as directly measured (Figure 4C) and the specific-rate data was then expressed in a time comparison manner (Figure 4D). This finding confirmed the blocked flux on the glycolysis pathway as shown by the transcriptional response of glycolysis. These data demonstrated that alterations of gene expression in iPSC-derived organoid differentiation can be characterized in the dynamics of metabolic flux, especially in glycolysis and the TCA cycle.

### Glycine, Serine, and Threonine Metabolism Contributes to DNA Methylation During Kidney Organoid Differentiation

**FIGURE 5:**
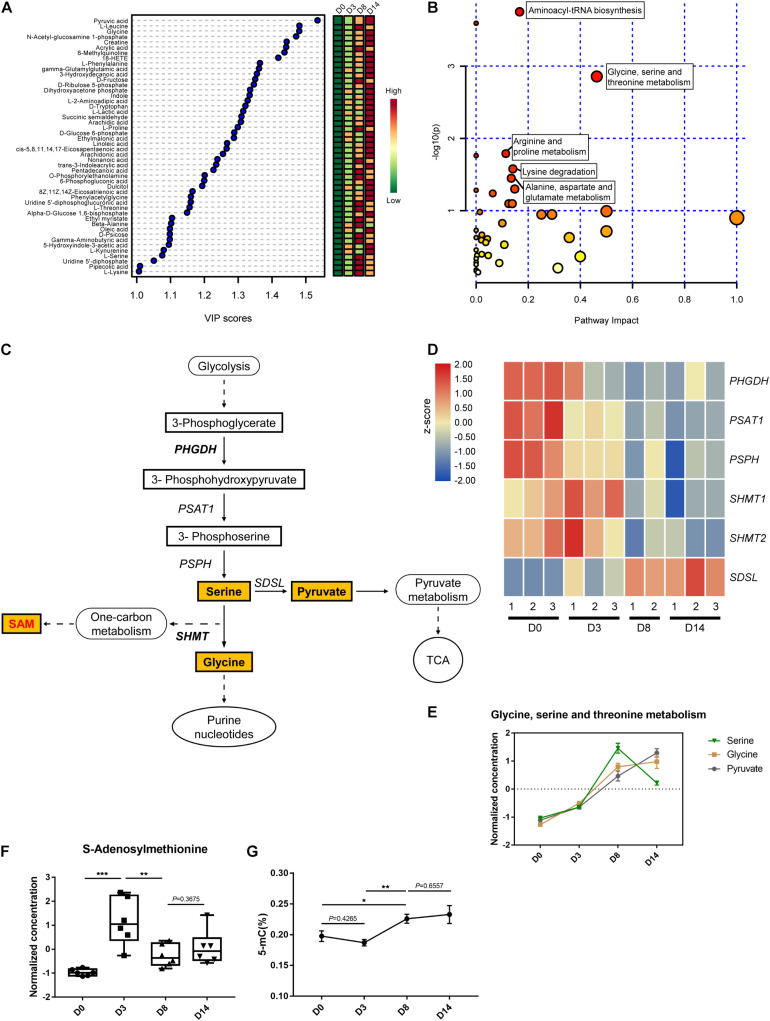
Metabolic profiling of the glycine, serine, and threonine metabolic pathway. **(A)** Variable importance in projection (VIP) scores of 48 intracellular metabolites above one calculated by the PLS-DA model. Colors represent average concentration from six biological replicates at each time point. **(B)** Pathway impact analysis (pathways with high *P*-value and/or high pathway impact are labeled). **(C)** Schematic of glycine, serine, and threonine metabolic and related pathways. Quantified metabolites are highlighted. **(D)** Heatmap of the relative expression level of genes related to serine metabolism. **(E)** The normalized and auto-scaled concentration of measured metabolites in glycine, serine, and threonine metabolism pathway. **(F)** The normalized and auto-scaled concentration of S-Adenosylmethionine (SAM) in different time points. **(G)** DNA methylation level during the kidney organoid differentiation process. The normalization of concentration of metabolites is described in methods. Data are presented as mean ± SEM from at least three biological replicates. *P < 0.05, **P < 0.01, and ***P < 0.001.

The supervised partial least squares-discriminant analysis (PLS-DA) was carried out to further investigate the most pronounced metabolic variations during kidney organoid differentiation. The variable importance for the projection (VIP) scores displaying the relative contributions of these representative metabolites indicated that VIP values for 48 out of 96 metabolites were above one (Figure 5A). These 48 metabolites indicated the most distinctive variations during differentiation, which hinted that these alterations in metabolites were related to the status of organoid formation. Further, pathway enrichment analysis of these 48 significant metabolites highlighted that glycine, serine, and threonine metabolism had the highest statistically significant top impact scores throughout the differentiation process (Figure 5B and Supplementary Table 1). Hence, we determined to characterize the contribution of serine-dependent metabolism in modulating kidney organoid differentiation.

It has been widely reported that the non-essential serine serves as a crucial precursor essential for the growth and survival of proliferating cells. As illustrated, the main pathway of glycine, serine, and threonine metabolism was branched from glycolysis at the node of 3-phosphoglycerate (3PG) (Figure 5C). As impaired activation in glycolysis, the genes expressed in the serine biosynthesis were downregulated, including phosphoglycerate dehydrogenase (PHGDH), phosphoserine aminotransferase (PSAT), and phosphoserine phosphatase (PSPH) encoded genes. The gene expression level was verified using qRT-PCR *via* selected markers *PHGDH* and *SDSL* (Supplementary Figure 3). The verified RNA-seq data were plotted in a heatmap (Figure 5D). Interestingly, this was accompanied by a striking accumulation of serine pool size in the early differentiation phase (D0–D8), and followed by a notable decline in the late phase (D8–D14). This was consistent with a direct measurement of the accumulation of serine activated during the generation of glycine (Figure 5E), which indicated the cell proliferation in the early phase of differentiation before the development of nephron structures.

Serine dehydratase (SDS) converts serine to generate pyruvate and contributes to the elevated TCA cycle *via* enrichment of pyruvate, which is in agreement with the metabolic profile observed during the late phase of differentiation (D8–D14) (Figures 5C–E). SAM functions as a methylation donor generated from *SHMT1/2* regulated serine one-carbon units branch and related to the metabolite-mediated epigenetic regulation. Thus, the concentration of SAM was measured using LC-MS. The data demonstrated that the peak level of SAM appeared on D3 of organoid differentiation, which was initiated from the base level of D0, and as the biphasic response observed in D8 and D14 (Figure 5F). This finding agreed with the level of SAM and correlated with the transcriptional regulation capability of serine hydroxymethyltransferase (SHMT) (Figure 5D). This gene expression pattern agreed with the quantified DNA methylation level, as the trend of 5-methyl-cytosine (5-mC) level during the kidney organoid differentiation process (Figure 5G). These findings were consistent with the variation of SAM, which suggested that the level of DNA methylation was potentiated before the formation of nephron structures while remaining stable in the later phase (Figures 5F,G).

## Discussion

**FIGURE 6:**
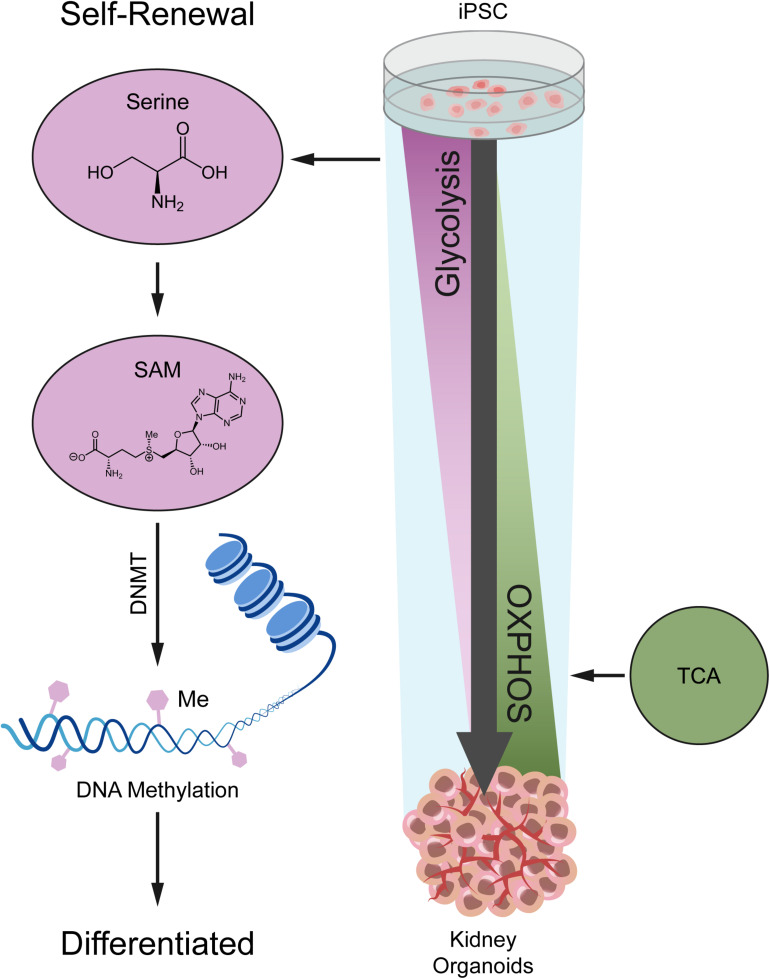
A model depicting the metabolic alteration from glycolysis in iPSC to OXPHOS in kidney organoids.

In this study, we investigated the metabolic dynamics during kidney organoid differentiation for 14 days, and we used untargeted metabolomics coupled with transcriptomics to determine the global portrait of the metabolic features (Figure 6). As we uncovered, nephrogenesis prefers mitochondrial OXPHOS to satisfy energy demand, consistent with the metabolic transition from glycolysis to OXPHOS during differentiation based on our kidney organoid differentiation model. These findings were also verified by using H1 human embryonic stem cell (hESC) line in independent assays (see Supplementary Figure 6).

Induced pluripotent stem cell differentiation followed the same trends with regard to metabolic status. Previous studies suggested that the metabolic status of iPSC differentiation actively dictated cell fate rather than acting as a marker of differentiation (Shyh-Chang et al., 2013; Zhang et al., 2014; Teslaa and Teitell, 2015). Our data demonstrated that the interplay between glycolysis, OXPHOS, and TCA cycle was highly dynamic during kidney organoid differentiation (Figures 3C,D, 4A). OXPHOS and TCA cycle are tightly coordinated, while glycolysis and TCA cycle are primary sources of metabolic precursors for biosynthesis and energy production, the balance among glycolysis, TCA cycle, and mitochondrial oxidative phosphorylation, which is essential for stem cell function. This alteration may be related to the rapid cell fate changes in the early stages (Figure 3D). The expression level of most genes related to glycolysis dramatically declined in the pluripotency stage (D0–D8) and gently decreased after nephron development. Notably, TCA generates the reducing equivalents (NADH and FADH_2_) to feed the mitochondrial respiratory chain, and OXPHOS is a highly efficient metabolic pathway to produce the majority of cellular energy in the form of ATP (Martínez-Reyes and Chandel, 2020). As a result of our experiments, we concluded that the kidney organoid differentiation was accompanied by the switch of cellular energy supply from glycolysis to OXPHOS.

Undoubtedly, the differentiation of kidney organoids derived from iPSCs was regulated epigenetically. In the literature, as reported, serine underpins the methionine cycle by providing one-carbon units for re-methylation of methionine and supporting *de novo* ATP synthesis to allow for the conversion of methionine to SAM (Maddocks et al., 2016). Moreover, SAM functions as a substrate for SAM-dependent DNA methyltransferases (DNMT), leading to methylation of DNA, which plays a crucial role in regulating epigenetic modifications (Yang and Vousden, 2016). We observed that serine was implied as a potential bioactive metabolite for distinguishing the process of kidney organoid differentiation. Serine accumulated continuously during early kidney organoid differentiation and decreased by day 14, which corresponded to a more mature state (Figures 5D–G). This period of differentiation was consistent with the varied levels of SAM and subsequently influenced the quantified 5-mC level. These were consistent with previous studies that have reported that the serine metabolism gene (*Psat1*), whose promoter was bound by the core pluripotency factors (OCT4, NANOG, and SOX2), was able to activate transcription and influence mouse ESC (mESC) differentiation, while maintaining a *Psat1* level that was essential for mESC self-renewal and pluripotency (Hwang et al., 2016). In contrast, during disease, the serine synthesis pathway’s upregulation supports cell proliferation and metastasis related to epigenetic regulation (Kim and Park, 2018; Zeng et al., 2019). Collectively, this finding unveiled that methylation acted as a fundamental mechanism in epigenetic regulation, and was strongly associated with serine metabolism.

The iPSC-derived organoids have been applied to many disease modeling studies (Lancaster and Huch, 2019). Utilization of patient-specific iPSCs to validate the functional single nucleotide polymorphisms (SNPs) or other non-coding regions (using organoids as the mini-tissue system) can face difficulties especially when determining phenotypes (Sun and Ding, 2017; Warren et al., 2017; Doss and Sachinidis, 2019; Rowe and Daley, 2019). A similar scenario can be more predominant in organoid-based compound toxicity screening, as multicellular heterogeneity might mask the impact of conventional approaches. Metabolomics is broadly acknowledged as the omics discipline closest to the phenotype and is yielding important new insights into several critical biological and physiological processes. Using the metabolomic insight to understand the organoid-based disease modeling or drug screening significantly consolidates the connection between transcriptomics and metabolomics to define the featured cross-disciplinary biomarkers or dynamic pattern. This approach opens the door for a more delicate and accurate readout that reflects the physiological and pathophysiological conditions.

## Materials and Methods

### Ethics Statement

All experiments were carried out with the guidelines set by the Human Subject Research Ethics Committee at Guangzhou Institutes of Biomedicine and Health (GIBH) and the Chinese Academy of Sciences (CAS), and the Committee approved the experiments (approval number GIBH-IRB07-2017039).

### hiPSC Generation

The human iPSCs used in this work were generated from urine-derived cells (UCs), which were derived from a healthy donor. The generation protocol was initiated from Xue et al. (2013) and modified as described previously (Fan et al., 2017; Sun et al., 2020b). Briefly, UCs were cultured in REGM:MEF (1:1) medium (UC medium). About 1–2 × 10^6^ primary UCs were separated by trypsin treatment (0.25% Trypsin/0.5 mM EDTA, Gibco, Grand Island, NE, United States) and electroporated with episomal plasmids using Amaxa Basic Nucleofector Kit for primary mammalian epithelial cells (T-020 program, Lonza, Basel, Switzerland) according to manufacturer’s instructions. Six (6) μg of the pEP4EO2SET2K vector containing the OCT4, SOX2, KLF4, and SV40LT genes and 4 μg of the pCEP4 vector carrying the miR302–367 were co-transfected into the UCs. The nucleofected UCs were seeded onto Matrigel (Corning, NY, United States) pre-coated 6-well culture plates in UC medium and replaced with mTeSR1 medium (STEMCELL, Vancouver, Canada) supplemented with 4i (4i: A83–01 (0.5 μM), thiazovivin (0.5 μM), PD0325901 (1 μM), and CHIR99021 (3 μM)) on the next day. After 11 days, the medium was changed to mTeSR1 without 4i. The iPSC colonies were manually picked at around day 20 and subsequently expanded as individual iPSC lines. A series of characterizations for the established iPSC lines were performed, including AP staining, karyotyping, teratoma formation, and analyses of pluripotency marker expression by qRT-PCR and immunocytochemistry (data not shown).

### Organoid Formation

The hiPSCs and H1 hESCs were maintained on a 10-cm Matrigel-coated dish to about 80% confluence before the initiation of differentiation. On day 0, cells were washed twice with PBS (HyClone, Logan, UT, United States), dissociated using dispase (1 mg/mL; Gibco), and then washed three more times with PBS and scraped with a cell lifter. Next, cells were gently pipetted into small clumps and resuspended in BPEL (supplemented with 8 μM CHIR99021, 3.3 mM Y27632, and 1 mM β-mercaptoethanol). Cell suspensions were evenly distributed into a 6-well ultra-low attachment plate (Corning, NY, United States) and cultured undisturbed until half the medium was replaced using BPEL combined with CHIR99021 (8 μM) on day 2. On day 3, embryoid bodies (EBs) formed, and all of them were transferred to a 50-mL tube and washed twice in DMEM. After that, EBs were resuspended in Stage II culture medium and distributed evenly to the 6-well ultra-low attachment plate. Half the Stage II medium was replaced every 2 days during organoid differentiation up until day 14. Preparation of BPEL and Stage II medium was performed as described in Przepiorski et al. (2018).

### RNA Extraction, cDNA Synthesis, and qRT-PCR

Total RNA in the cells was isolated using TRIzol (Invitrogen, Carlsbad, CA, United States) and extracted using HiPure Universal RNA Mini Kit (Magen, Guangzhou, China). About 1 μg of total RNA was used for cDNA synthesis using GoScript Reverse Transcription Mix with Oligo (dT) (Promega, Madison, WI, United States). qRT-PCR was performed with SsoAdvanced SYBR Green Supermix (Bio-Rad, Irvine, CA, United States) in an ABI StepOnePlus real-time PCR machine. The relative gene expression was calculated by the ddCt method and normalized to the internal standard gene *GAPDH*. All of the samples were measured in triplicates. Primer sequences used for qRT-PCR are listed in Supplementary Table 2.

### Immunofluorescence Analysis

The organoids were fixed with paraformaldehyde (4%) in PBS for 30 min at room temperature (RT), washed three times with PBS, and then incubated with sucrose (30% w/v) in PBS overnight at 4°C. Organoids were then embedded in optimal cutting temperature (OCT) compound (Tissue-Tek, Torrance, CA, United States) to make frozen blocks and cut into 10-μm sections. The sections were washed three times with PBS (5 min each time), then incubated in blocking buffer (0.2% Triton X-100 and 10% FBS in PBS) for 2 h at RT. After blocking, the sections were incubated with primary antibodies in antibody dilution buffer (10% FBS in PBS) overnight at 4°C. The following primary antibodies and dilutions were used: NPHS1 (AF4269-SP, R&D systems, 1:200), CD31 (555444, BD, 1:200), LTL-biotin-conjugated (FL-1321, Vector Labs, 1:100), CDH1 (610181, BD, 1:200), LRP2 (ab76969, Abcam, 1:200), UMOD (SAB1400296-50UG, Sigma, 1:200), GATA3 (MAB6330, R&D, 1:200), and MEIS1/2/3 (39796, Active Motif, 1:200). After washing in PBS three times (5 min each time), the sections were incubated with Alexa-Fluor secondary antibodies (Invitrogen) and diluted in PBS for 1 h at RT, then washed three times in PBS. Nuclei were counterstained with DAPI for 10 min. Images were obtained using a Zeiss LSM 710 confocal microscope (Oberkochen, Germany).

### Transmission Electron Microscopy

The organoids were fixed with electron microscopy fixation buffer consisting of glutaraldehyde (2.5%), paraformaldehyde (2%), and phosphate buffer (PB, 0.1 M, pH 7.4) at 4°C. Postfixation was performed using 1% OsO_4_ in PB (0.1 M) for 1 h at 4°C, dehydrated in a graded series of ethanol solutions, and embedded in epoxy resin. Ultra-thin sections (70 nm) were cut and stained with uranyl acetate and lead citrate. Sections were then imaged using FEI Tecnai G2 Spirit transmission electron microscope (Hillsboro, OR, United States).

### Bioinformatic Analysis for Transcriptome Samples

Cell samples from four different time points (D0, D3, D8, and D14) during the differentiation were transferred into TRIzol (Invitrogen) and stored at −80°C. The RNA extraction, quality detection, library establishment, and sequencing on a NovaSeq 6000 system (Illumina, San Diego, CA, United States) were carried out by Tianjin Novogene Technology Co., Ltd. (Tianjin, China).

For data analysis, Bowtie2 (v2.2.5) was used to align the filtered reads to the reference human genome (UCSC GRCh38/hg38), and RSEM (v1.2.22) was customized to count the gene expression level. The subsequent analysis was performed using the iDEP.91 tool^1^ (Ge et al., 2018). Only genes with more than 0.5 counts per million (CPM) in at least one sample were retained. CPMs were calculated by normalizing the read counts by the total counts per sample. The data were normalized by EdgeR and transformed by log_2_ (CPM+4) as recommended by iDEP.91. Moreover, PCA analysis and k-Means clustering of gene expression were then performed on iDEP.91 as well. David Bioinformatics Resources 6.8 (DAVID)^2^ was used for GO annotation and KEGG pathway enrichment analysis (Huang et al., 2009). The expression patterns of the gene sets of interest were defined by the R package Mfuzz (Kumar and E Futschik, 2007). The heatmap of gene expression data was plotted using TBtools v1.068, and the expression level was transformed by the z-score (Chen et al., 2020).

### Untargeted Metabolomic Analysis for Intracellular Metabolites

Samples used for harvesting intracellular metabolites were collected and quenched as established in Sellick et al. (2011). Quenched samples were then transferred to 1.5-mL tubes, and water (200 μL) was added into each sample after freeze-drying. The samples were vortexed for 30 s, homogenized at 45 Hz for 4 min with silica beads, and sonicated for 5 min in an ice-water bath (three times). Next, 20 μL of homogenate solution was isolated for protein quantification by the BCA Protein Quantification Kit (Vazyme, Nanjing, China). A volume of 180 μL acetonitrile was mixed with the residue 180 μL homogenate solution. Subsequently, the mixture was vortexed, homogenized, and sonicated as mentioned above for intracellular metabolite extraction, and then incubated (at −20°C for 1 h) and centrifuged (at 12,000 × *g* and 4°C for 15 min). The obtained supernatants were transferred to LC-MS vials and stored at −80°C until metabolomics analysis was performed. The quality control (QC) sample was prepared by mixing an equal aliquot of the supernatants from all of the samples.

LC-MS/MS analysis was conducted using a UHPLC system (UltiMate 3000, Thermo Fisher Scientific, Waltham, MA, United States) equipped with a UPLC HSS T3 column (2.1 mm × 100 mm, 1.8 μm, Waters, Milford, CT, United States) coupled to Q Exactive HF-X (Orbitrap MS, Thermo Fisher Scientific). The mobile phase A was 0.1% formic acid in water for positive ESI mode, 5 mmol/L ammonium acetate in water for negative ESI mode, and the mobile phase B was acetonitrile. The elution gradient was set as follows: 0 min, 1% B; 1.5 min, 1% B; 12 min, 99% B; 19 min, 99% B; 21 min, 1% B; 25 min, 1% B. The flow rate was 0.3 mL/min. A 10 μL sample was used for injection. The QE mass spectrometer was used for its ability to acquire MS/MS spectra on an information-dependent basis (IDA), and the acquisition software (Xcalibur 4.0.27, Thermo Fisher Scientific) enabled the collection of the data. ESI source conditions were set as follows: Sheath gas flow rate (45 Arb), Aux gas flow rate (15 Arb), Capillary temperature (400°C), Full ms resolution (60,000), MS/MS resolution (15,000), Collision energy (20/40/60 eV) in NCE model, and Spray Voltage: 4.0 kV (positive) or −3.6 kV (negative).

The raw metabolomics data were then processed by Compound Discoverer 3.0 software (Thermo Fisher Scientific). All acquired MS data were searched against available databases for metabolite annotation, including mzCloud, ChemSpider, and mzVault. Statistical analysis of metabolomics data was performed using MetaboAnalyst 4.0^3^ (Chong et al., 2019). Data were normalized by sample-specific total protein content and scaled by autoscaling (mean-centered and divided by the standard deviation of each variable). Unsupervised PCA and supervised PLS-DA were carried out to discover significant variations among the groups. The variable importance for the projection (VIP) scores of the first principal component in the PLS-DA model (VIP > 1.0) was used to select distinguishing metabolites during the cultivation, and one-way analysis of variance (ANOVA) was used to validate the selection (*P*-value < 0.01). Pathway enrichment analysis of the potential distinguishing metabolites was performed in MetaboAnalyst’s pathway analysis module.

### Quantification of Extracellular Metabolites

The concentration of residual glucose and secreted lactate in the culture medium was determined using an M-900 cell culture biochemical analyzer (SIEMAN, Shenzhen, China) according to the manufacturer’s instructions. 300 μL of the supernatant of each medium sample during the cultivation was inserted into the analyzer. Measurements were based on a direct reading of glucose and lactate in the medium by the enzyme sensors. After cells were lysed in RIPA lysis buffer (Beyotime, Shanghai, China), and total protein was measured by a BCA Protein Quantification Kit (Vazyme). The concentration of extracellular metabolites from different differentiation phases was normalized by the cell protein content.

### Quantification of Global DNA Methylation

Genomic DNA of the cells was extracted using the TIANamp Genomic DNA Kit (TIANGEN, Beijing, China). DNA methylation was measured using the colorimetric MethylFlash Global DNA Methylation 5-mC ELISA Easy Kit (Epigentek, Farmingdale, NY, United States) according to the manufacturer’s instructions.

### Statistical Analysis

GraphPad Prism 7 (GraphPad Software, San Diego, CA, United States) was used to conduct statistical analyses. Data are expressed as mean ± standard error of the mean (SEM) of independent measurements or assays (at least *n* = 3 replicates were considered). Statistical significance was evaluated using unpaired Student’s *t*-test with *P* < 0.05 for comparisons except for special circumstances described.

## Data Availability Statement

The required RNA-seq data have been deposited in the Sequence Read Archive (SRA) with BioProject accession code PRJNA692767.

## Author Contributions

MG and XZ initiated this study and coordinated the project. QW and XZ conceived the project. QW and SZ designed the research. QW and YX performed the experiments. QW, SZ, and YS analyzed the data. CY analyzed the RNA-seq data. PL assisted in LC-MS detection. HL performed the TEM analysis. QW and XZ co-wrote the final text. QW, YS, and WG contributed to review and editing. AP and AD provided the technical assistance. YG, AD, MG, and XZ revised and approved the final manuscript. All authors contributed to the article and approved the submitted version.

## Conflict of Interest

The authors declare that the research was conducted in the absence of any commercial or financial relationships that could be construed as a potential conflict of interest.

## Footnotes

1 http://bioinformatics.sdstate.edu/idep/

2 https://david.ncifcrf.gov

3 https://www.metaboanalyst.ca/
