# Supplementary material for: The Dynamics of Metabolic Characterization in iPSC-Derived Kidney Organoid Differentiation via a Comparative Omics Approach
Source: Front Genet. 2021 Feb 10;12:632810. doi: 10.3389/fgene.2021.632810 (PMC7902935; doi:10.3389/fgene.2021.632810)
Supplement: Supplementary file 1 [file Data_Sheet_1.docx]

Supplementary Material


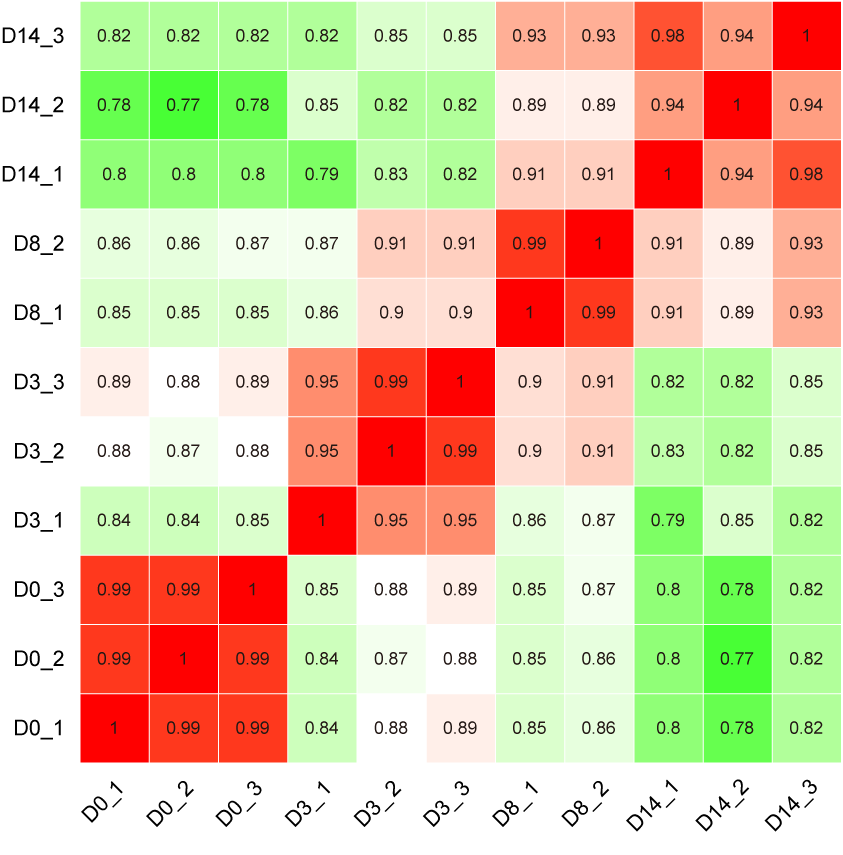


**Supplementary Figure 1.** Correlation coefficients of RNA-seq samples from different differentiation phases (D0, D3, D8, and D14).


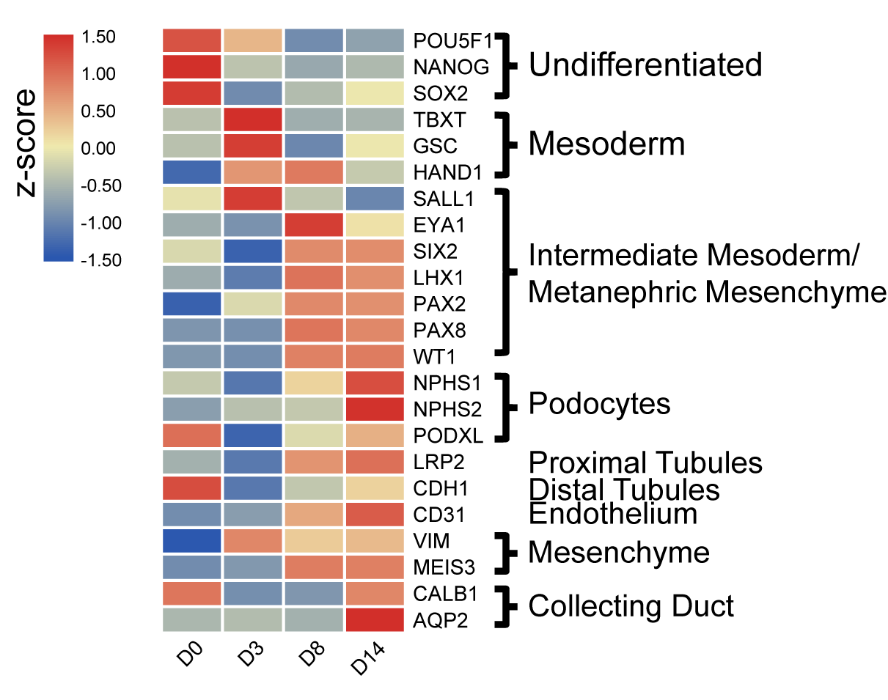


**Supplementary Figure 2.** The relative expression level of selected marker genes of kidney development during differentiation is based on the RNA-seq results.


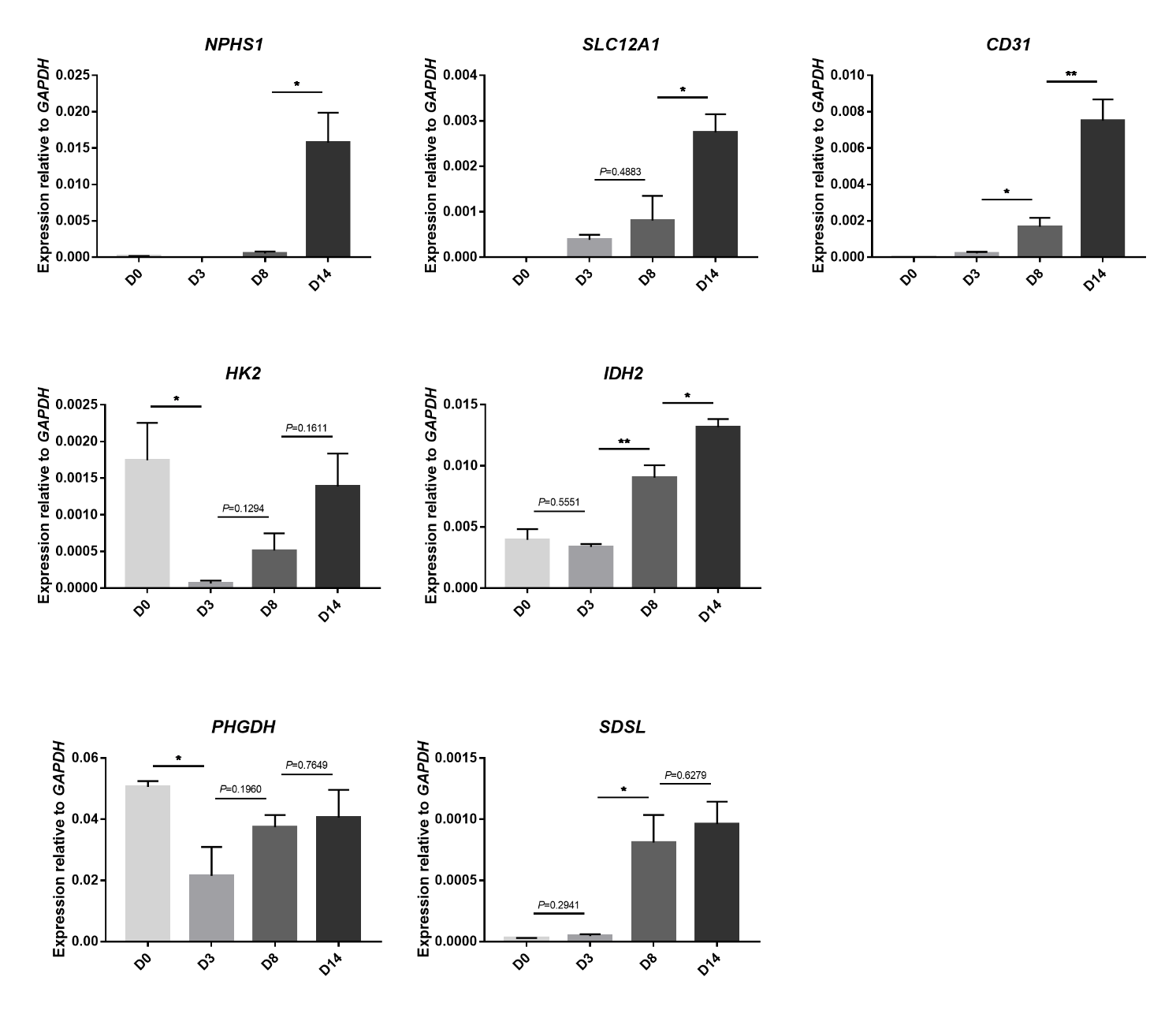


**Supplementary Figure 3.** qRT-PCR verification of selected marker genes related to RNA-seq data shown in Figure 3D and Figure 5D. **P*<0.05, ***P*<0.01.


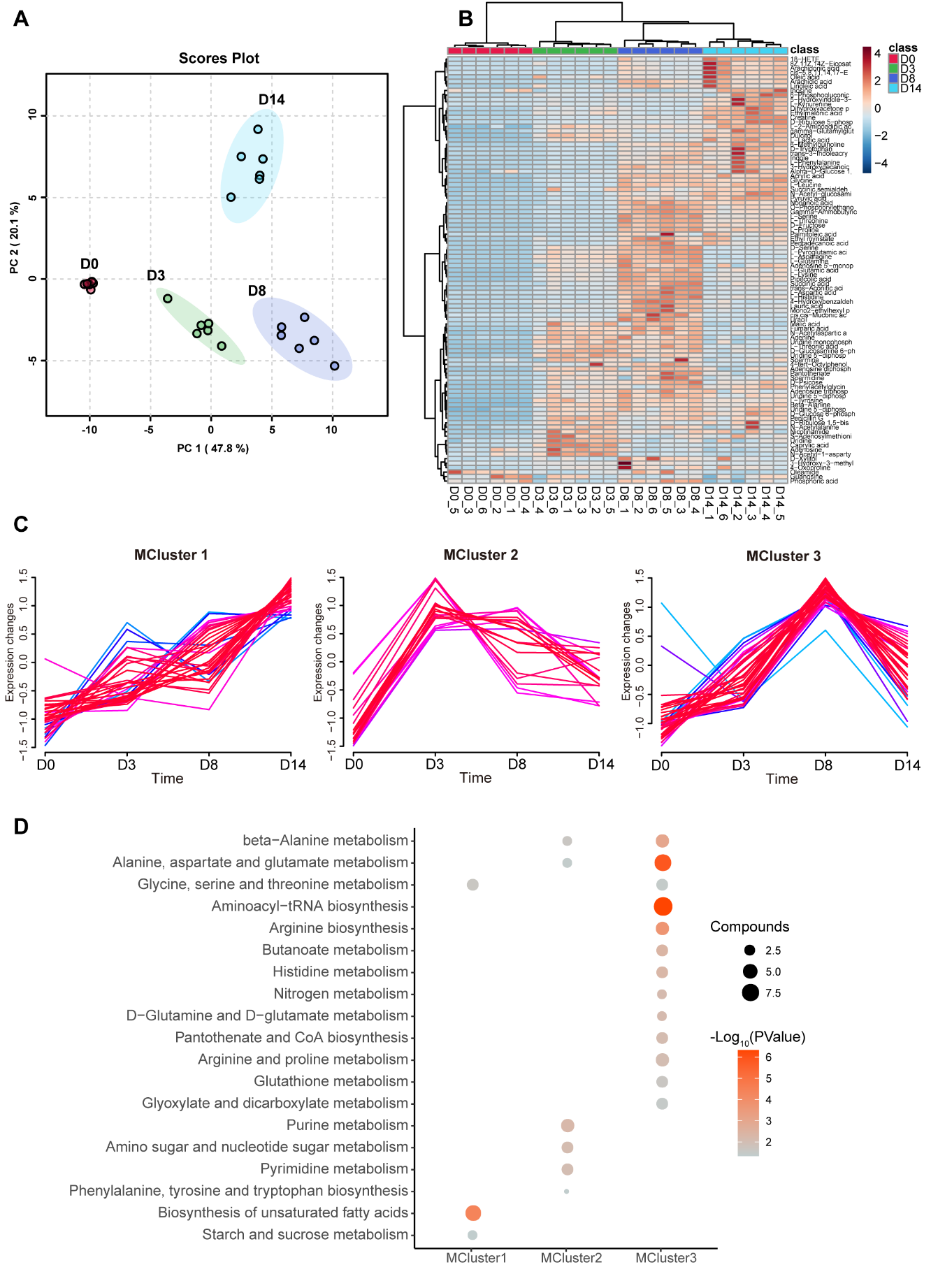


**Supplementary Figure 4.** Metabolomic data analysis at different phases of differentiation. (A) Scores plot for samples in PCA analysis. (B) Heatmap of overview metabolite features among different samples. (C) Clusters of intracellular metabolites at different phases of differentiation. (D) KEGG pathway enrichment analysis based on clustering analysis. Pathways with a *P*-value below 0.05 are shown.


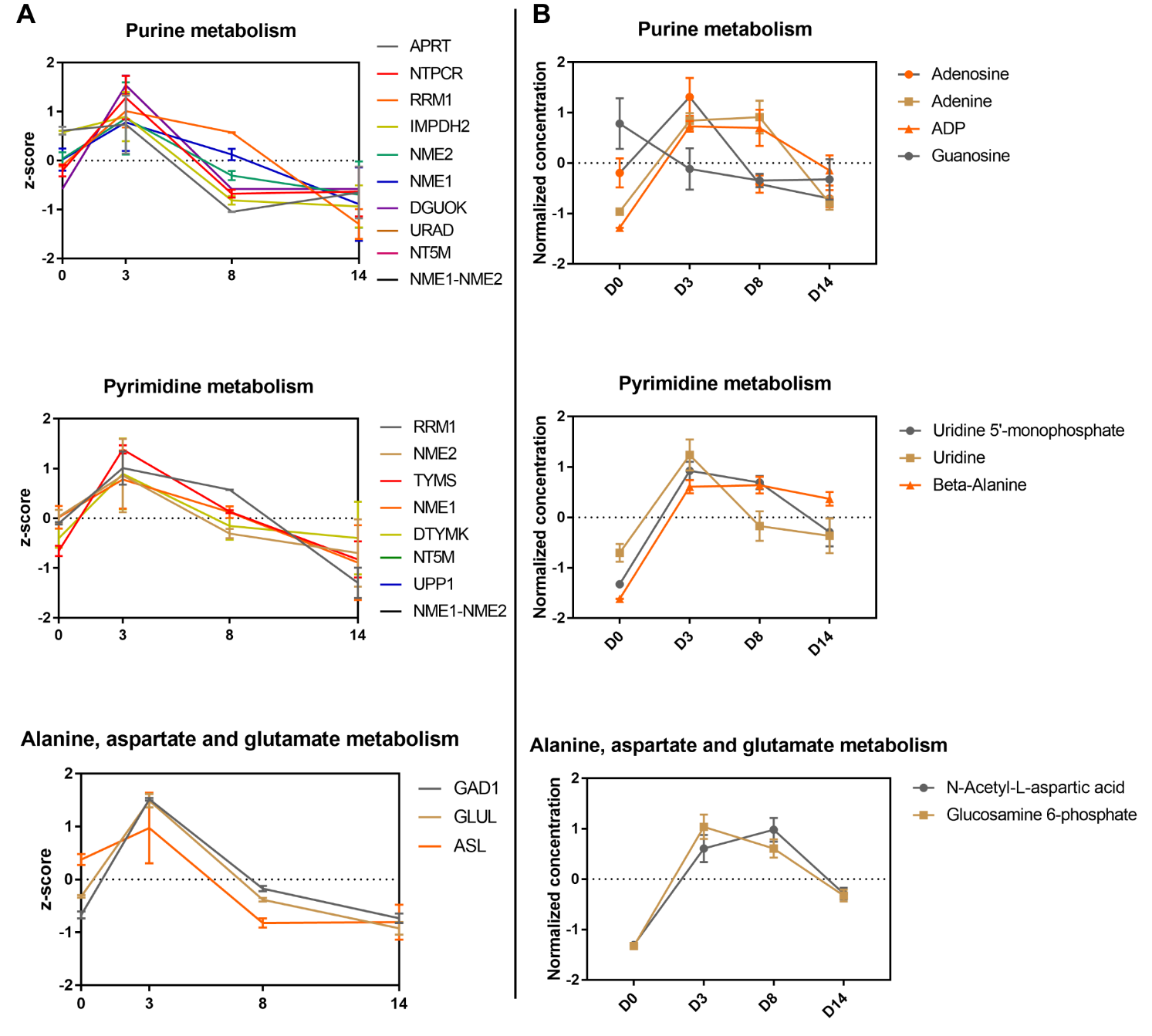


**Supplementary Figure 5.** Alteration of expressed genes (A) and metabolites (B) related to different metabolic pathways. Arbitrary units are used to show the trend changes in both parameters.


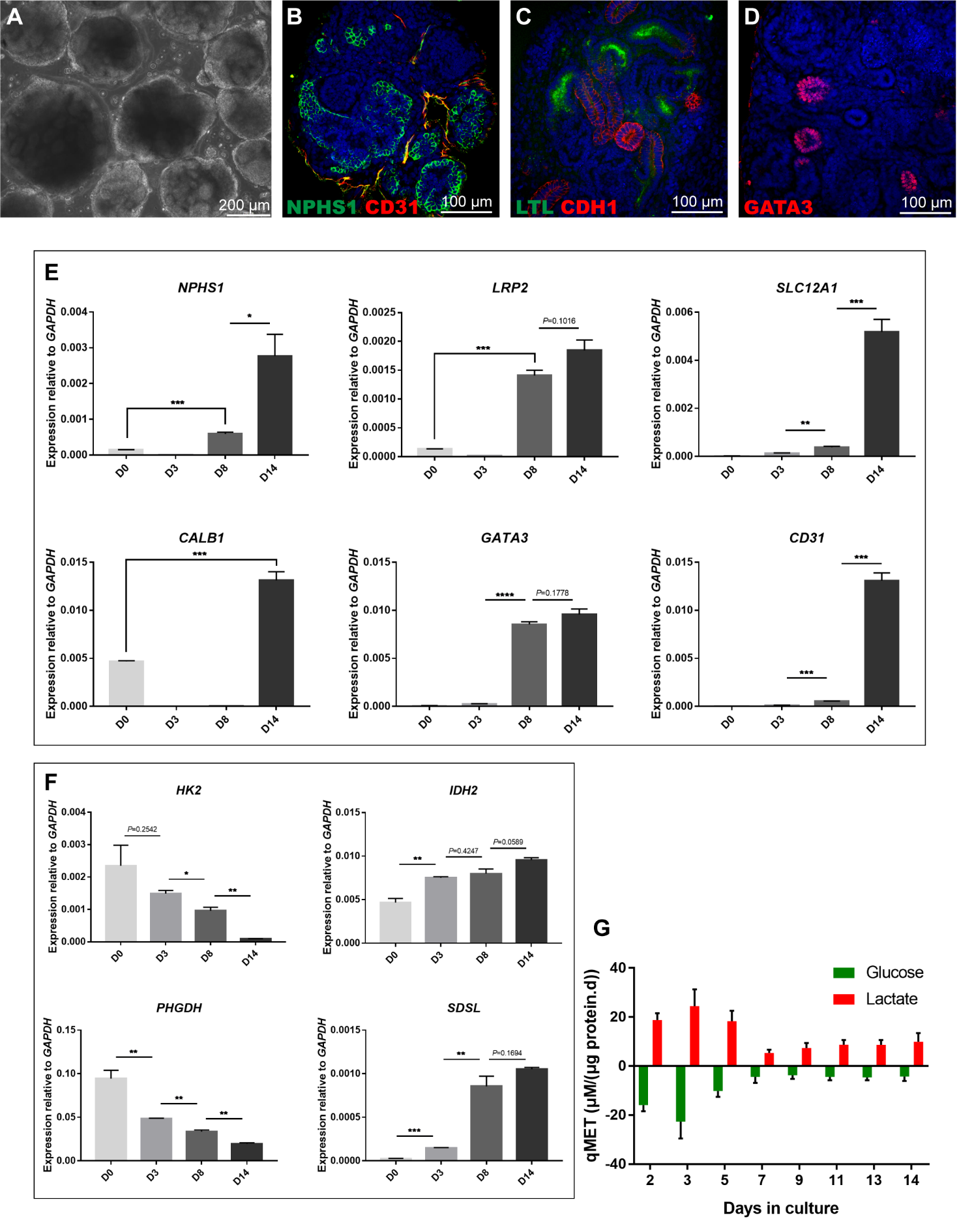


**Supplementary Figure 6.** Independent assays of characterizing kidney organoids derived from H1 human embryonic stem cell (hESC) line. (A) Brightfield image of organoids at day 14. (B-D) Immunofluorescent staining of frozen sections of day 14 organoids, labeled for NPHS1+ podocytes and CD31+ endothelial cells, LTL+ proximal tubules and CDH1+ distal tubules, and GATA3+ collecting duct structures. Nuclei are stained with DAPI. (E-F) qRT-PCR analysis for selected differentiated marker genes (E) and metabolic-related genes (F) during the differentiation at day 0, 3, 8, and 14. (G) Specific consumption rates of glucose (green column) and specific generation rate of lactate (red column) at different time points. Data are presented as mean±SEM from at least three biological replicates. **P*<0.05, ***P*<0.01, ****P*<0.001, *****P*<0.0001.

**Supplementary Table 1.** Details of pathway enrichment based on 48 significant metabolites calculated by the PLS-DA model (pathways with *P*-value above 0.05 are not shown).

|  | Total | Hits | *P*-value | Impact |
| --- | --- | --- | --- | --- |
| Aminoacyl-tRNA biosynthesis | 48 | 7 | 0.00018 | 0.16667 |
| Biosynthesis of unsaturated fatty acids | 36 | 6 | 0.00025 | 0 |
| Glycine, serine and threonine metabolism | 33 | 5 | 0.00139 | 0.46284 |
| Arginine and proline metabolism | 38 | 4 | 0.01623 | 0.11377 |
| Valine, leucine and isoleucine biosynthesis | 8 | 2 | 0.01729 | 0 |
| Lysine degradation | 25 | 3 | 0.02638 | 0.14085 |
| Alanine, aspartate and glutamate metabolism | 28 | 3 | 0.03558 | 0.13462 |

**Supplementary Table 2.** List of qRT-PCR primers used in this paper.

| **Gene** | **Forward primer (5′-3′)** | **Reverse primer (5′-3′)** |
| --- | --- | --- |
| *GAPDH* | GGCATGGACTGTGGTCATGAG | TGCACCACCAACTGCTTAGC |
| *NPHS1* | AGTGTGGCTAAGGGATTACCC | TCACCGTGAATGTTCTGTTCC |
| *LRP2* | AAATTGAGCACAGCACCTTTGA | TCTGCTTTCCTGACTCGAATAATG |
| *SLC12A1* | AGTGCCCAGTAATACCAATCGC | GCCTAAAGCTGATTCTGAGTCTT |
| *CALB1* | TCCAGGGAATCAAAATGTGTGG | GCACAGATCCTTCAGTAAAGCA |
| *GATA3* | GCCCCTCATTAAGCCCAAG | TTGTGGTGGTCTGACAGTTCG |
| *CD31* | AACAGTGTTGACATGAAGAGCC | TGTAAAACAGCACGTCATCCTT |
| *HK2* | GAGCCACCACTCACCCTACT | CCAGGCATTCGGCAATGTG |
| *IDH2* | CGCCACTATGCCGACAAAAG | ACTGCCAGATAATACGGGTCA |
| *PHGDH* | CTGCGGAAAGTGCTCATCAGT | TGGCAGAGCGAACAATAAGGC |
| *SDSL* | GACGGCTGGGAGAATGTCC | ATGGCCGCATTGAAGCAGT |
